# A Systematic Literature Review of Packed Red Cell Transfusion Usage in Adult Extracorporeal Membrane Oxygenation

**DOI:** 10.3390/membranes11040251

**Published:** 2021-03-30

**Authors:** Thomas Hughes, David Zhang, Priya Nair, Hergen Buscher

**Affiliations:** 1 Department of Intensive Care Medicine, St Vincent’s Hospital, Sydney 2010, Australia; thomas.hughes@health.nsw.gov.au (T.H.); Priya.Nair@svha.org.au (P.N.); 2 Faculty of Medicine, University of New South Wales, Sydney 2052, Australia; davidzhang549@gmail.com

**Keywords:** ECMO, extracorporeal membrane oxygenation, extracorporeal life support, blood management, transfusion

## Abstract

Background: Blood product administration plays a major role in the management of patients treated with extracorporeal membrane oxygenation (ECMO) and may be a contributor to morbidity and mortality. Methods: We performed a systematic review of the published literature to determine the current usage of packed red cell transfusions. Predefined search criteria were used to identify journal articles reporting transfusion practice in ECMO by interrogating EMBASE and Medline databases and following the PRISMA statement. Results: Out of 1579 abstracts screened, articles reporting ECMO usage in a minimum of 10 adult patients were included. Full texts of 331 articles were obtained, and 54 were included in the final analysis. All studies were observational (2 were designed prospectively, and two were multicentre). A total of 3808 patients were reported (range 10–517). Mean exposure to ECMO was 8.2 days (95% confidence interval (CI) 7.0–9.4). A median of 5.6% was not transfused (interquartile range (IQR) 0–11.3%, 19 studies). The mean red cell transfusion per ECMO run was 17.7 units (CI 14.2–21.2, from 52 studies) or 2.60 units per day (CI 1.93–3.27, from 49 studies). The median survival to discharge was 50.8% (IQR 40.0–64.9%). Conclusion: Current evidence on transfusion practice in ECMO is mainly drawn from single-centre observational trials and varies widely. The need for transfusions is highly variable. Confounding factors influencing transfusion practice need to be identified in prospective multicentre studies to mitigate potential harmful effects and generate hypotheses for interventional trials.

## 1. Introduction

Extracorporeal membrane oxygenation (ECMO) is a rapidly evolving area of intensive care practice, with the potential to rescue patients with severe cardiac or respiratory failure who would almost certainly have died in earlier eras. However, there is a paucity of high-quality evidence to guide this invasive and resource-intensive therapy, and as such, much practice for avoiding ECMO-induced harm is guided by consensus, first principles and local policy.

The extracorporeal circuit exposes the entire blood volume to a large surface area of artificial material as well as significant shear forces, with resultant red cell damage and deranged activation of the coagulation pathways. Depending on the cannulation strategy, the site of vascular access may also be a point of significant blood loss. Therefore, hemorrhage, hemolysis and decreased red cell lifespan are ubiquitous in patients receiving ECMO. Further, thrombocytopenia and coagulopathies are common findings, either due to anticoagulant therapy, the underlying condition, or the circuit itself. Thus, patients receiving ECMO have a substantial transfusion requirement, with the attendant comorbidity and drain on blood bank resources. Management of anticoagulation during ECMO is an area of intense research, with recent systematic reviews [1,2] attempting to address the varying patterns of management. However, to date, transfusion practice has not been given the same attention.

Within broader intensive care practice, transfusion strategy has been guided by seminal trials such as TRIC [3] and the ensuing meta-analyses [4], whereby red cell transfusion triggers of 7 g/dL are now commonplace for most patient groups, with separate consideration given to patients with active bleeding or high risk of ischemia, such as due to flow-limiting atherosclerotic lesions. It is not well established where optimal transfusion management of the highly heterogeneous ECMO population sits within the similarly heterogeneous ICU population; however, it is believed that ECMO patients have an increased risk of bleeding and exposure to blood products, but this has not been quantified.

The goal of this review is to better characterise historical and recent red cell transfusion practice within adult ECMO patients.

## 2. Materials and Methods

The study followed the Preferred Reporting Items for Systematic Reviews and Meta-Analyses (PRISMA) statement [5].

### 2.1. Search Strategy

We searched multiple electronic databases (Medline and EMBASE) to identify all potentially relevant publications in English reporting transfusion burden in ECMO patients between 1996 and 2016. The search strategies are specified in Table S1.

### 2.2. Study Inclusion and Exclusion

Studies were included if they reported on 10 or more patients aged 18 years or older, where ECMO was used for support in the ICU. For mixed studies, 20% or fewer patients aged under 18 was considered acceptable. ECMO used solely as a substitute for traditional cardiopulmonary bypass in the operating theatre was not accepted. Concurrent use of an intra-aortic balloon pump was acceptable. Studies reporting on other mechanical cardiovascular support modalities, such as a ventricular assist device concurrently with ECMO, were only included when a discrete ECMO-only group, meeting all other criteria, was present. Articles were excluded if the quantity of red cell transfusions was not reported. As a minimum, studies needed to state red cell transfusion amounts per ECMO run, per day or for the whole cohort. For consistency, product usage was required to be by component (e.g., red cells) rather than aggregated, and, similarly, data reported as mL/kg were excluded. Studies reporting outcomes related to a single event in the ECMO run (e.g., decannulation), or a limited time frame rather than the full ECMO run, were not included. Finally, when otherwise valid but chronologically overlapping cohorts from the same institution were encountered, only the largest cohort was included.

### 2.3. Data Extraction, Quality Assessment and Analysis

One author (TH) performed a full-text review and data extraction, with oversight from a senior colleague (HB). The primary outcome recorded was daily or total transfusion usage per patient. Secondary outcomes of interest were transfusion practice by ECMO modality, indication, duration, and survival status. Where available, cannulation strategy, membrane type, anticoagulation target (either activated clotting time (ACT) or activated partial thromboplastin time (aPTT)); prespecified transfusion triggers and the fraction of patients not transfused were also recorded. This information was tabulated and processed with Microsoft Excel. Quality assessment of the selected full-text papers was performed using the Newcastle-Ottawa Quality Assessment Scale [6].

To facilitate comparison, transfusion volumes were converted to units of red cells (300 mL). Mean values for a variable were estimated, where necessary, using median-to-mean formulae outlined by Wan [7] for sets with a range or interquartile value. Meta-analysis and forest plot generation were then performed using R with the *meta* package [8], with the random effect size assumption after assessment of heterogeneity through I^2^. Values without standard deviation data were reported as median/interquartile range.

## 3. Results

### 3.1. Search Results and Characteristics of Included Studies

The original search identified 1577 citations, and two further citations were added during the review of references from a full-text assessment. Exclusion was recorded based on a single criterion, although studies were frequently rejected on several grounds. The most common exclusions were pediatric focus (510), fewer than 10 patients reported (330) and ECMO use (or lack thereof) not meeting the criteria in the methods section above (185). The remaining exclusions were for absent abstracts, duplicate citations, review articles and nonhuman/ex-vivo reports.

**Figure 1:**
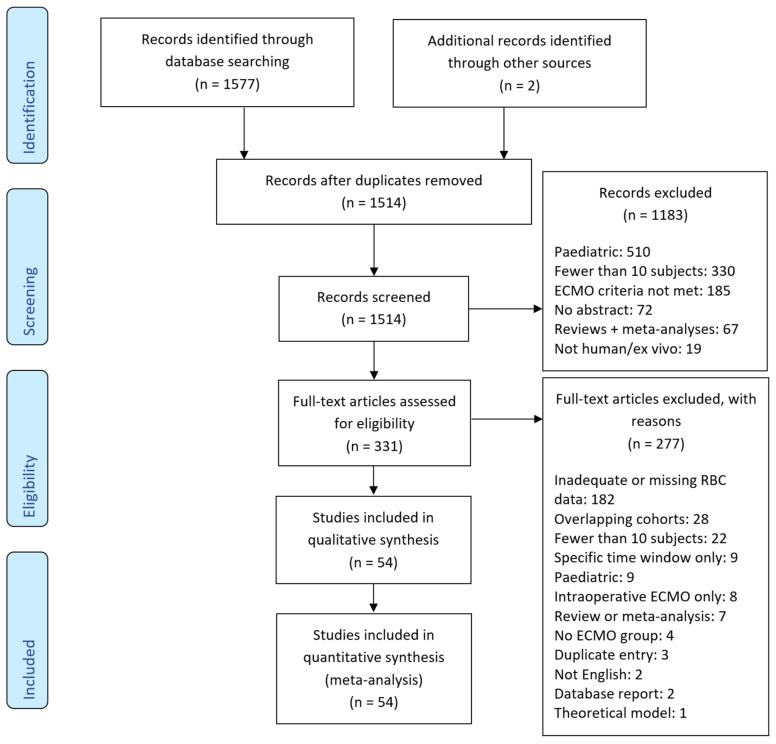
CONSORT diagram.

Full-text assessment was performed on the remaining 331 publications. The most frequent reason for exclusion was inadequate or missing data for RBC transfusion (182 studies). Other exclusions are detailed in the consort diagram (Figure 1). A further 28 studies were not able to be included due to the inclusion of a study from the same institution in an overlapping recruitment period and with a larger cohort. All included studies are summarised in Table 1 (54 studies).

### 3.2. Methodological Quality

Two studies were designed prospectively, with the remainder reporting retrospective reviews of institutional databases. One study reported on 15 centres [53], another included three hospitals [30], and all others were single-centre. All papers were scored on the Newcastle-Ottawa scale as cohort studies, with a maximum possible score of 9. The median score was 7 (IQR 6–7; Table S2).

### 3.3. Patient and ECMO Characteristics

Fifty-four studies reported a transfusion dose during ECMO, with a total of 3808 patients. Four studies each had patients under 18 years [39,53,55,61] (3 of 15, 3 of 68, 3 of 38 and 1 of 23 patients, respectively), whilst the remainder were entirely adult cohorts. Other characteristics are described in Table 1.

Exclusively postcardiotomy cohorts were represented in 8 studies (1078 patients), with one paper reporting on patients requiring ECMO for primary cardiac graft dysfunction [50] and the remainder reporting outcomes after a variety of cardiac surgical procedures. Twenty-seven studies (1349 patients) covered exclusively nonsurgical patients, predominantly patients receiving venovenous (VV) ECMO due to ARDS and other severe acute respiratory failure, although 6 of these 27 were venoarterial (VA) cohorts related to postinfarction cardiogenic shock or ECMO-facilitated CPR.

Survival to hospital discharge was available in 41 studies (2984 patients), with a median of 50.8% (IQR 40.0–64.9%). Survival of ECMO alone was reported in 28 studies (1635 patients), with a median of 65.2% (CI 56.1–69.6%).

Peripheral cannulation was the dominant strategy, present in 2756 of 3375 (81.6%) patients with available data. This was broadly distributed, with 28 studies reporting rates of 100% and a further 7 studies reporting rates above 80%. The remaining 11 studies, where data were provided, had peripheral cannulation rates between 39–80%, whilst 9 studies did not report their cannulation strategy.

Centrifugal pumps were most common (40 studies). The remainder were accounted for by roller pumps (*n* = 5), a mixture (*n* = 2), other pump designs (*n* = 1) or not specified (*n* = 6).

Where reported, most membranes used were poly-methyl pentene (PMP; *n* = 33). Polypropylene (*n* = 5), silicon (*n* = 3) and combinations of membrane types (*n* = 3) were the rest (not specified in 9 studies). No cohort commencing after 2006 reported a membrane-type other than PMP.

Thresholds for the administration of blood products were given in less than half of the included studies. Nineteen studies specified a hemoglobin concentration (median 8 g/dL, range 7–15, IQR 8–10), whilst 10 specified a hematocrit threshold (median 28%, range 24–35, IQR 28–30). To facilitate comparison, hematocrit targets were converted to hemoglobin concentration by dividing by three.

Platelet targets were provided in 22 studies (median transfusion trigger 50,000/µL, range 20,000–100,000, IQR 50,000–75,000). Only 5 studies mentioned targets for fibrinogen concentration (range 1–3 g/L), and 2 reported an INR threshold.

Nineteen studies reported whether transfusion was universal in their cohort, with a median of 5.6% (IQR 0–11.3%) not receiving red cell support during ECMO. This was broadly distributed, with 5 studies reporting a universal need for transfusion, whilst other studies reported rates as high as 60% [44] and 67% [17] of freedom from red cell transfusion.

### 3.4. Reported Complications

Hemorrhage as the direct cause of death had a median incidence of 2% (IQR 0–6%, 16 studies with 890 patients). Intracranial hemorrhage occurred in 4% (IQR 2–7%, 25 studies with 2207 patients). Procedural intervention for bleeding was reported in 16 studies (1308 patients) with a median frequency of 35% (IQR 11–46%). Major bleeding, as per the heterogeneous definitions thereof in the 16 studies (651 patients) reporting it, occurred in a median of 30% of ECMO patients (IQR 18–45%).

Ischemic stroke was reported in 20 papers (1810 patients), with a median incidence of 5% (IQR 2–10%); 4 of these publications reported no patients with strokes. Limb ischemia and DVT were frequently reported together; the aggregated outcome was noted in a median of 12% of patients (IQR 6–20%, 28 studies with 2067 patients). Intracardiac clot incidence was reported in only 3 papers (76 patients) with rates of 4, 5 and 15 percent. Circuit failure (or requirement for circuit change as a surrogate for impending failure) occurred in a median of 9% of patients (IQR 5–15%) in the 20 studies (1642 patients).

Renal failure requiring dialysis support frequently occurred (median 49% [IQR 38–58%]; 28 studies, 2197 patients).

### 3.5. Transfusion Rates

**Figure 2:**
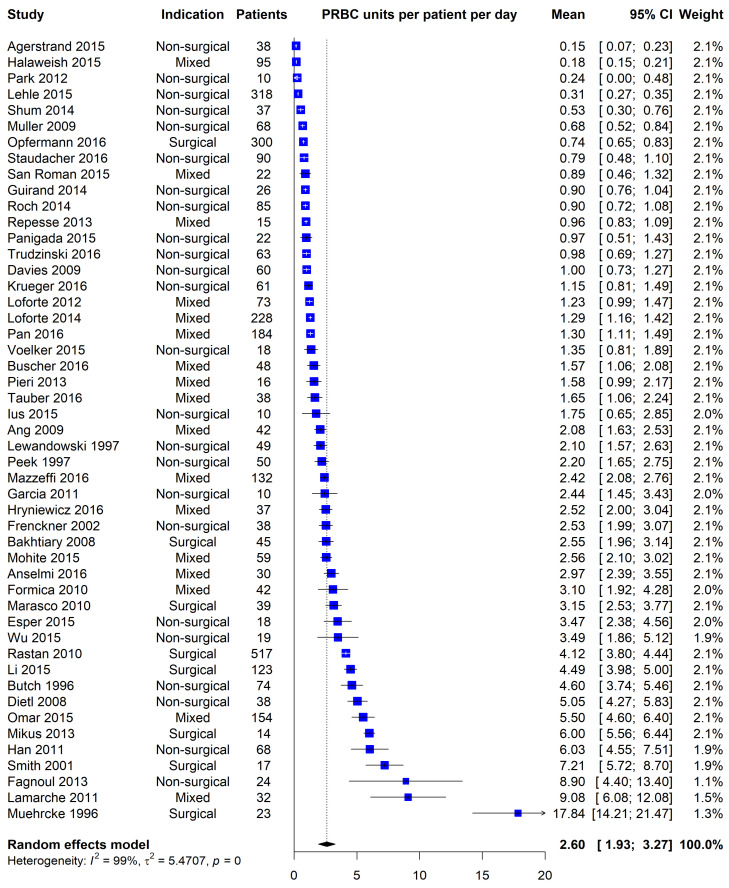
Forest Plot-all included studies.

**Figure 3:**
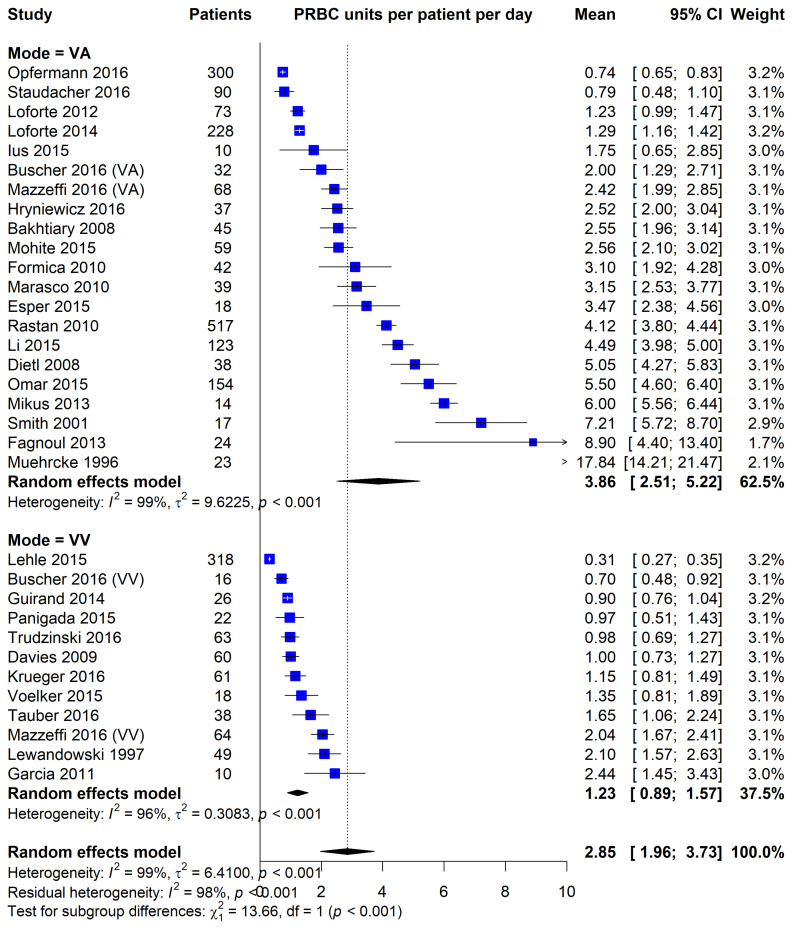
Forest plot comparing VV and VA modality.

The meta-analysed transfusion data is presented in Table 2 and Table 3, and Figure 2 and Figure 3.

VV patients received significantly fewer transfusions per ECMO day (1.23 units (0.89–1.57) versus 3.86 (2.51–5.22), *p* < 0.001) but not per ECMO run (19.3 (10.4–28.1) versus 18.3 (14.2–22.4)) when compared to patients treated with VA ECMO. Studies with postcardiotomy patients (5.56 (2.20–8.93) versus 1.93 (1.26–2.59), *p* = 0.04) and with a >10% rate of central cannulation (4.53 (2.31–6.76) versus 1.74 (1.24–2.25), *p* = 0.02) had twice as many transfusions per ECMO day compared to other studies. Studies reporting an above-median survival rate also reported significantly less need for PRBC transfusions (1.65 (1.08–2.23) versus 3.82 (2.23–5.42), *p* = 0.001). If a below-median transfusion trigger was used, the associated number of PRBC transfusions was significantly less (1.41 (0.86–1.97) versus 2.39 (1.67–3.10), *p* = 0.005). However, no significant association was seen between the upper anticoagulation target (either ACT or APTT groups) and the frequency of transfusions. A major bleeding event rate above the median was also not associated with more PRBC transfusions.

## 4. Discussion

To the best of our knowledge, this is the first study to provide a synopsis of red cell transfusion practice in published ECMO literature. Transfusion practices and thresholds vary widely by patient indication, institution, and country, in part due to the dearth of quality trial data to date. Similarly, practices have varied significantly over time-early editions of the Extracorporeal Life Support Organization’s guidelines (the “Red Book”) [63], which called for hemoglobin targets of 15 g/dL, whilst most studies in our review that commenced after 2006 transfused for hemoglobin levels less than 8–10 g/dL.

In the 51 studies included in our pooled effect calculation, patients received a mean of 2.60 units of PRBCs per day of ECMO support. However, the distribution of values from our studies was wide, ranging from 0.15–17.8 units per patient per day, an unsurprising finding given the diverse range of patient cohorts sampled. The subgroup comparisons performed begin to suggest some of the drivers for this heterogeneity, with our findings in keeping with other published data from smaller data sets and meta-analyses addressing complications in specific subgroups.

VA ECMO predicted higher transfusion rates in several single-centre studies where a comparison was made with VV [10,14,64], and our study suggested an approximately three-fold increase in red cell use for VA patients. A 2019 meta-analysis [65] suggested central cannulation was associated with higher rates of in-hospital death, reoperation for bleeding complications and transfusion, in keeping with the association seen in our analysis, where groups with exclusive or very high rates of peripheral cannulation had a significantly lower transfusion burden. Postcardiotomy ECMO use also appears to be associated with greater frequency of transfusion; however, this is an almost-exclusively VA ECMO cohort, with higher rates of central cannulation than most other ECMO indications, as well as an expected higher frequency of bleeding events and coagulation disturbances due to the nature of the operations and of exposure to intraoperative cardiopulmonary bypass. As such, there is a significant confounding effect present that our study is not powered to disentangle.

Several studies included in this review have drawn associations between increased transfusions and poorer survival in ECMO patients [14,64,66,67] as well as in other ICU populations such as post cardiac surgery [68], while our work suggests higher transfusion rates in cohorts with below-median survival. The direction and strength of this association are uncertain, as the transfusion of any allogeneic blood product comes with well-recognised immunologic and nonimmunologic risks. Conversely, the requirement for blood transfusion may be a signal of underlying adverse events (especially hemorrhage or hemolysis) that are themselves more directly likely to lead to death.

The use of PMP membranes versus earlier membrane technology (based on silicon or polypropylene) appeared to show a lower transfusion rate, but this finding fell short of statistical significance. This is out of keeping with published experience, starting with early cohorts of patients managed with PMP membranes [69]. The difference reported in other series has been attributed to decreased membrane surface area leading to lower rates of contact activation of clotting processes, a lower priming volume and heparin-coated surfaces. All included studies commencing after 2006 used PMP membranes exclusively. Other changes in ECMO equipment over our study period include a shift toward centrifugal pumps and heparin-bonded circuits, which are thought to decrease red-cell trauma [23] and coagulation activation, which may all have contributed to this finding.

Adoption of a lower transfusion threshold was associated with a lower red cell transfusion rate. One single-centre trial found the implementation of a more restrictive transfusion protocol for postcardiotomy VA ECMO patients led to a drop of 45% in red cell units transfused per ECMO run [70].

Significant heterogeneity in transfusion targets was seen, which is not unexpected; one published international survey [71] of critical care clinicians found the greatest variation in transfusion thresholds was for ECMO patients. Centres with higher ECMO volumes have reported lower thresholds for transfusions from clinician surveys [72]. In our review, most studies commenced after 2009 had a threshold of 10 g/dL or lower. This evolution is likely to be driven by a variety of factors, including greater familiarity with ECMO management as well as the growth of critical care literature finding noninferiority of lower transfusion thresholds in other patient groups, such as patients with sepsis [73], GI bleeding [74] and after cardiac surgery [75]. These trials have been influential on a more restrictive transfusion practice being adopted in the broader ICU population, and it is not unreasonable to think this change has leached into ECMO management as well.

One area where our study showed weaker associations was anticoagulation targets and bleeding complications, with neither variable showing a robust association with transfusion rates. This finding may be driven by the smaller number of studies included. For anticoagulation, the spread of anticoagulation targets was relatively narrow and across two noncomparable modalities (ACT and aPTT), which may limit the ability to distinguish a real finding. Further, anticoagulation targets are only a surrogate for the achieved degree of anticoagulation (which would be expected to be a better predictor of bleeding events and, thus, transfusion) and do not reliably account for other commonly found derangements of coagulation function in ECMO patients. Several single-centre reports [13,76,77,78] suggest that lower anticoagulation targets or anticoagulation-free ECMO is feasible and is associated with lower rates of bleeding and transfusion. For bleeding, the lower number of included patients, as well as the lack of a standardised definition of bleeding, likely confounded the result, as, from first principles, a higher rate of major bleeding would be expected to predict a greater need for transfusion. This could be further explored by using standardised criteria such as those proposed by the Bleeding Academic Research Consortium [79].

### 4.1. Data Quality

All included publications were observational cohort studies—some included a case-control design, but the data of interest were best described as a cohort in how it was extracted and incorporated into the analysis. Overall, the quality of papers was relatively consistent—most were retrospective cohorts where the outcomes of interest were readily demonstrated (ECMO exposure and transfusion outcomes), and, furthermore, papers that were inadequate in these areas generally did not meet all inclusion criteria; more variability was seen in follow-up arrangements, such as whether survival after ICU or hospital discharge was tracked.

Many studies were excluded for not publishing transfusion data, even in circumstances of discussing bleeding on ECMO or aggregating all product types in their data. Similarly, the heterogeneity of the patient population studied was also broad in terms of indication, with its implication for likely blood product requirements. However, given the role of ECMO as a therapy at the end of a final common pathway of cardiac or respiratory deterioration, this is a strength of our data set.

### 4.2. Limitations and Sources of Error

The heterogeneity of our data set, as well as the heterogeneity of reporting red cell use and relevant complications such as bleeding, is a distinct limitation for drawing detailed conclusions about cause and effect. It is unknown whether our cohort is representative of the global ECMO population, which has likely evolved and diversified as ECMO has become a more accepted and viable support option. Equally, our results could be skewed by publication bias as it is possible that studies with particularly high or low transfusion rates might choose not to highlight this data. This is partly counteracted by the inclusive nature of the study. The only criteria needed for inclusion was to report a red cell transfusion rate, which is, thus, the most robust quantitative finding of this study, along with the comparison of VA and VV patients.

Conversely, variable reporting or lack of stratification of other outcomes of interest, such as transfusion triggers, and ECMO indications (e.g., many cohorts had a mix of indications) and complications, limited the depth of interpretation behind predictors of red cell use, and the subgroup analyses we have performed are best viewed as associations worthy of further research.

### 4.3. Implications for Future Research

Future research into ECMO transfusion practice should ideally be prospective and multicentre, with standardisation of reporting blood product usage and outcomes such as hemorrhagic complications. Such studies are currently on the way for VA-ECMO (NCT03714048) and VV-ECMO (NCT03815773). Future interventional studies addressing modifiable factors such as transfusion triggers, equipment, cannulation strategies and anticoagulation would be a significant improvement on the current state of knowledge.

## 5. Conclusions

This study demonstrated a substantial transfusion requirement during ECMO and demonstrated significant heterogeneity of transfusion practice. The evidence is largely drawn from single-centre retrospective observational data, which limits interrogation of confounding factors influencing transfusion practice. The impact of mode, indication, equipment, and anticoagulation and transfusion triggers should be further investigated in prospective multicentre studies to better identify potentially harmful aspects of ECMO transfusion practice and generate hypotheses for the evaluation in future interventional trials for this resource-intensive therapy.

## Figures and Tables

**Table 1:** Summary of included studies.

Author/Year (Reference)	Location	Study Period Start and End Year	N	ECMO Type (VA/VV/Not Spec)	ECMO Days (Mean +/−SD)	Transfusion Trigger (g/dL or HCt %)	% Not Transfused PRBC on ECMO	PRBC/Day (Mean +/−SD)	Survival to Discharge (%)	Study Type	Brief Description
Anselmi 2016 [9]	France	2005–2014	30	27/3/0	8.9 (+/−7.3)	24%	0.0%	2.97 (+/−1.63)	50.0%	R. obs	Mixed—cardiogenic shock after heart transplant (47%) or cardiotomy (40%) and small number of respiratory failures—cohort report on use of recombinant factor VIIa
Buscher 2016 [10]	Australia	2009–2010	48	32/16/0	8.0 (+/−7.0)	8 g/dl	8.3%	1.57 (+/−1.79)	69.0%	R. obs	Mixed—cardiogenic shock of all causes, including eCPR and severe respiratory failure (mostly ARDS but 3 post-transplant)
Czobor 2016 [11]	USA	2012–2014	25	25/0/0	NR		8.0%		40.0%	R. obs	Nonsurgical—cardiogenic shock and eCPR—cohort report on predictive utility of SOFA score
Hryniewicz 2016 [12]	USA	2012–2013	37	37/0/0	4.7 (+/−2.3)		8.1%	2.52 (+/−1.61)	64.9%	R. obs	Mixed cardiogenic shock post-AMI (18), cardiotomy (5), decompensated failure (6)
Krueger 2016 [13]	Germany	2011–2015	61	0/61/0	12.0 (+/−6.5)	10 g/dL		1.15 (+/−1.35)		R. obs	Nonsurgical—respiratory failure, principally ARDS—cohort review for outcomes of anticoagulation with VTE prophylaxis only in VV ECMO patients
Mazzeffi 2016 [14]	USA	2010–2013	132	68/54/0	8.0 (+/−6.7)			2.42 (+/−1.97)	50.8%	R. obs	Mixed—cardiogenic shock, mostly postcardiotomy (38) and ARDS (54)—cohort review for predictors of bleeding events
Opfermann 2016 [15]	Austria	2001–2014	300	300/0/0	6.1 (+/−4.8)			0.74 (+/−0.79)	51.7%	R. obs	Surgical—cardiogenic shock postcardiotomy—cohort review for predictors of survival
Pan 2016 [16]	Australia	2010–2014	184	128/56/0	7.0 (+/−4.9)			1.30 (+/−1.33)	73.4%	R. obs	Mixed—cardiogenic shock of varying causes, including postcardiotomy, post-transplant and severe respiratory failure of multiple causes—cohort review for predictors of elevated plasma-free Hb
Staudacher 2016 [17]	Germany	2010–2013	90	90/0/0	2.2 (+/−2.7)	8 g/dL	67.8%	0.79 (+/−1.51)	24.4%	R. obs	Nonsurgical—cardiogenic shock after arrest or AMI—cohort comparison of outcomes of antiplatelet therapy vs. none
Tanaka 2016 [18]	USA	2010–2014	84	84/0/0					41.7%	R. obs	Mixed—mostly cardiogenic shock, small postcardiotomy group—cohort review of predictors for vascular access complications
Tauber 2016 [19]	Austria	2010–2012	38	26/12/0		8.5 g/dL	0.0%	1.65 (+/−1.87)		Prosp. obs	Mixed—cardiogenic shock and severe respiratory failure—cohort review for predictors of higher transfusion requirement
Trudzinski 2016 [20]	Germany	2010–2015	63	0/63/0	22.4 (+/−17.4)	7 g/dL (or ScvO2<65%)		0.98 (+/−1.17)	66.7%	R. obs	Nonsurgical—half ARDS, half chronic lung disease awaiting transplant
Agerstrand 2015 [21]	USA	2010–2012	38	4/34/0	9.2 (+/−3.5)	7g/dL	36.8%	0.15 (+/−0.25)	73.7%	R. obs	Nonsurgical—respiratory +/− cardiac failure due to ARDS of varying aetiologies—cohort report on restrictive approach to transfusions
Esper 2015 [22]	USA	2007–2013	18	18/0/0	3.3 (+/−2.2)		5.6%	3.47 (+/−2.36)	66.7%	R. obs	Nonsurgical—cardiogenic shock after AMI
Halaweish 2015 [23]	USA	2002–2013	95	18/66/11	15.5 (+/−13.4)			0.18 (+/−0.16)	63.2%	R. obs	Mixed—mainly respiratory failure (75); also cardiogenic shock (14) and eCPR (6)—cohort comparison of roller and centrifugal pumps, only duration >5days
Ius 2015 [24]	Germany	2012–2014	10	10/0/0	10.2 (+/−4.2)		10.0%	1.75 (+/−1.78)	50.0%	R. obs	Nonsurgical—acute on chronic respiratory failure—cohort of VV ECMO requiring conversion to VV-A
Lehle 2015 [25]	Germany	2009–2014	318	0/318/0		8 g/dL		0.31 (+/−0.36)		R. obs	Nonsurgical—mixed respiratory failure cohort (pneumonia, trauma, acute on chronic lung disease, pulmonary haemorrhage)—cohort report on predictors of ECMO-associated haemolysis
Li 2015 [26]	China	2011–2012	123	123/0/0	4.3 (+/−3.7)	30%		4.49 (+/−2.88)	34.1%	R. obs	Surgical—cardiogenic shock post-cardiotomy
Mohite 2015 [27]	UK	2010–2014	59	59/0/0	8.9 (+/−5.1)			2.56 (+/−1.81)		R. obs	Mixed—cardiogenic shock (decompensated heart failure, postcardiotomy, post-AMI)—cohort comparison of outcomes between sedated and “awake” ECMO patients
Omar 2015 [28]	USA	2007–2013	154	126/28/0	5.6 (+/−6.6)			5.50 (+/−5.71)	33.1%	R. obs	Mixed—mainly cardiogenic shock (cardiomyopathy, eCPR, AMI, postcardiotomy, heart transplant, PE) with smaller group respiratory failure and lung transplant—cohort report on predictors of mortality on ECMO, including plasma-free Hb
Panigada 2015 [29]	Italy	2011–2013	22	0/22/0	9.0 (+/−5.5)			0.97 (+/−1.09)		Prosp. obs	Nonsurgical—respiratory failure due to ARDS/COPD or bridge to lung transplant—cohort report comparing clinical, lab and CT findings for oxygenator thrombosis
Poss 2015 [30]	Germany	2012–2013	15	15/0/0			26.7%		66.7%	R. obs 3ctr	Nonsurgical—cardiogenic shock, mostly post-AMI, some myocarditis—cohort comparison of ECMO vs. i-Cor assist device
San Roman 2015 [31]	Argentina	2011–2014	22	9/13/0	5.1 (+/−4.3)		0.0%	0.89 (+/−1.02)	68.2%	R. obs	Mixed—cardiorespiratory failure in pre- and postoperative lung transplant plus group of non-transplant respiratory failure
Voelker 2015 [32]	Germany	2009–2011	18	0/18/0	21.7 (+/−30.0)	7 g/dL		1.35 (+/−1.16)	61.1%	R. obs	Nonsurgical—respiratory failure (pneumonia, trauma, other)—cohort report on restrictive transfusion approach
Wu 2015 [33]	Taiwan	2008–2014	19	10/9/0	7.0 (+/−4.8)			3.49 (+/−3.62)	68.4%	R. obs	Nonsurgical—respiratory failure (trauma-associated ARDS)
Guirand 2014 [34]	USA	2001–2009	26	0/26/0	9.3 (+/−9.5)			0.90 (+/−0.36)	57.7%	R. obs	Nonsurgical—respiratory failure (trauma-associated ARDS)
Loforte 2014 [35]	Italy	2006–2012	228	228/0/0	10.8 (+/−9.2)	28%	0.0%	1.29 (+/−1.03)	63.2%	R. obs 2 ctr	Mixed—cardiogenic shock, mostly postcardiotomy (118), transplant failure (37), post-AMI (27), decompensated heart failure (40) and myocarditis (6)
Roch 2014 [36]	France	2009–2013	85	8/77/0	9.7 (+/−4.5)	10 g/dL		0.90 (+/−0.86)	43.5%	R. obs	Nonsurgical—respiratory failure (ARDS)
Shum 2014 [37]	Hong Kong	2009–2013	37	13/24/0	5.5 (+/−2.3)		0.0%	0.53 (+/−0.72)	73.0%	R. obs	Nonsurgical—mostly pneumonia, smaller cohort myocarditis—cohort report on regional citrate anticoagulation for haemodialysis access via ECMO circuit
Fagnoul 2013 [38]	Belgium	2012–2012	24	24/0/0	1.6 (+/−2.1)	7 g/dL	12.5%	8.90 (+/−11.25)	25.0%	Prosp. obs	Nonsurgical—eCPR
Michaels 2013 [39]	USA	2009–2010	15	7/8/0	9.8 (+/−1.0)			3.90 (NR)	60.0%	R. obs	Nonsurgical—respiratory failure (H1N1 influenza)
Mikus 2013 [40]	Italy	2007–2011	14	14/0/0	9.0 (+/−13.8)	28%	0.0%	6.00 (+/−0.84)	42.9%	R. obs	Surgical—postcardiotomy cardiogenic shock—cohort report on CentriMag pump
Pieri 2013 [41]	Italy	2009–2012	16	13/3/0	6.0 (+/−4.0)	8 g/dL28%		1.58 (+/−1.20)		R. obs	Mixed—cardiogenic shock (mixed primary CS or postsurgical) or ARDS—cohort report on use of phosphorylcholine-coated oxygenator
Repesse 2013 [42]	France	2006–2011	15	11/4/0	17.3 (+/−8.9)	24%		0.96 (+/−0.26)		R. obs	Mixed—cardiogenic shock (mixed primary CS or postsurgical) or ARDS—cohort report of use of recombinant factor VIIa for refractory bleeding on ECMO
Loforte 2012 [43]	Italy	2007–2011	73	73/0/0	10.9 (+/−7.6)	28%	0.0%	1.23 (+/−1.04)	45.2%	R. obs	Mixed—cardiogenic shock, mostly postcardiotomy (50/73), 12/73 post-AMI and 8/73 post-heart transplant
Park 2012 [44]	Brazil	Not reported	10	2/8/0	9.2 (+/−9.4)		60.0%	0.24 (+/−0.39)	40.0%	R. obs	Nonsurgical—mixed respiratory failure (mostly pneumonia)—cohort of patients from commencement of ECMO service in this hospital
Garcia 2011 [45]	USA	2009–2009	10	0/10/0	20.0 (+/−15.0)	35%		2.44 (+/−1.60)	60.0%	R. obs	Nonsurgical—mixed respiratory failure (ARDS, advanced chronic respiratory disease pending lung Tx)—cohort report on ambulating VV ECMO patients
Han 2011 [46]	South Korea	2006–2009	68	59/9/0	5.3 (+/−6.6)	35%		6.03 (+/−6.23)		R. obs	Nonsurgical—cardiogenic shock or respiratory failure (ARDS)—comparison of nafamostat vs. heparin for anticoagulation during ECMO; large cohort of eCPR (41/68)
Lamarche 2011 [47]	Canada	2000–2009	32	32/0/0	2.2 (+/−2.0)			9.08 (+/−8.66)		R. obs	Mixed—cardiogenic shock, primary or associated with cardiac surgery, some eCPR-comparison of Impella vs. ECMO
Formica 2010 [48]	Italy	2002–2009	42	42/0/0	7.9 (+/−5.3)	30%		3.10 (+/−3.90)	38.1%	R. obs	Mixed—cardiogenic shock, primary or associated with cardiac surgery, 2/42 massive PE
Kanji 2010 [49]	Canada	2002–2006	50	50/0/0	2.9 (+/−2.6)	10 g/dL		12.38 (NR)		R. obs	Mixed—cardiogenic shock, primary or associated with cardiac surgery—comparison of peripheral vs. central cannulation with respect to transfusion and bleeding events
Marasco 2010 [50]	Australia	2000–2009	39	39/0/0	6.8 (+/−2.6)	8 g/dL		3.15 (+/−1.99)		R. obs	Surgical—post-heart transplant primary graft failure
Rastan 2010 [51]	Germany	1996–2008	517	517/0/0	3.3 (+/−2.9)			4.12 (+/−3.67)	24.8%	R. obs	Surgical—postcardiotomy cardiogenic shock
Ang 2009 [52]	Singapore	2003–2006	42	37/5/0	6.5 (+/−3.2)	10 g/dL		2.08 (+/−1.49)	26.2%	R. obs	Mixed—pre- and post-cardiac surgery, myocarditis, PE, severe respiratory failure
Davies 2009 [53]	Australia	2009–2009	68	5/65/0	10.7 (+/−6.1)			0.68 (+/−0.67)		R. obs 15 ctr	Nonsurgical—H1N1 pneumonia and other viral ARDS
Muller 2009 [54]	Germany	2006–2008	60	0/60/0	9.0 (+/−6.1)	8 g/dL		1.00 (+/−1.06)	45.0%	R. obs	Nonsurgical—mixed severe respiratory failure (pneumonia/trauma/aspiration/sepsis/other)
Bakhtiary 2008 [55]	Germany	2003–2006	45	45/0/0	6.4 (+/−4.5)			2.55 (+/−2.03)	28.9%	R. obs	Surgical—postcardiotomy cardiogenic shock—mixed indications (CABG/valves/LVAD, 2/45 post heart transplant)
Dietl 2008 [56]	USA	1994–2006	38	38/0/0	5.6 (+/−2.6)			5.05 (+/−2.45)	60.5%	R. obs	Nonsurgical—Hantavirus cardiopulmonary syndrome
Frenckner 2002 [57]	Sweden	1995–2002	38	0/0/38	17.0 (+/−12.9)			2.53 (+/−1.70)		R. obs	Nonsurgical—mixed severe respiratory failure (pneumonia/trauma/PE/aspiration/other)
Smith 2001 [58]	Australia	1995–1998	17	17/0/0	4.1 (+/−2.1)	10 g/dL		7.21 (+/−3.13)	41.2%	R. obs	Surgical—postcardiotomy cardiogenic shock
Lewandowski 1997 [59]	Germany	1989–1995	49	0/49/0	23.1 (+/−19.7)	15 g/dL		2.10 (+/−1.90)	55.1%	R. obs	Nonsurgical—respiratory failure (ARDS)
Peek 1997 [60]	UK	1989–1995	50	2/48/0	8.6 (+/−7.4)	14 g/dL	4.0%	2.20 (+/−2.00)	66.0%	R. obs	Nonsurgical—respiratory failure (ARDS/pneumonia/asthma)—mixed cohort
Author/year (reference)	Location	Study period start and end year	N	ECMO type (VA/VV/not spec)	ECMO days (mean +/−SD)	Transfusion trigger (g/dL or HCt %)	% not transfused PRBC on ECMO	PRBC/day (mean +/−SD)	Survival to discharge (%)	Study type	Brief description
Butch 1996 [61]	USA	1988–1994	74	0/0/74	10.9 (+/−10.9)	14 g/dL	1.4%	4.60 (+/−3.77)	45.9%	R. obs	Nonsurgical—respiratory failure (ARDS/pneumonia/asthma)—mixed cohort (infection, trauma, post-solid organ transplant)
Muehrcke 1996 [62]	USA	1992–1994	23	23/0/0	2.4 (+/−1.5)			17.84 (+/−8.88)	31.8%	R. obs	Surgical—postcardiotomy cardiogenic shock

Abbreviations: ECMO—extracorporeal membrane oxygenation, VA—venoarterial, VV—venovenous, HCt—hematocrit, PRBC—units of packed red blood cells, AMI—acute myocardial infarction; ARDS—acute respiratory distress syndrome; eCPR—ECMO-facilitated cardiopulmonary resuscitation; LVAD—left ventricular assist device; NR—data not reported; SOFA—Sequential Organ Failure Assessment (score); study types: retrospective (R) or prospective (P) observational.

**Table 2:** Baseline characteristics of included studies.

Variable		Finding (95% Confidence Range)	Number of Papers (Patients) Included	Cochrane’s Q Test	I^2^ Test of Heterogeneity	*p*-Value for Comparison
Baseline Characteristics						
Age (years)		48.9 (46.3–51.5)	53 (3786)	2128	98%	n/a
Gender (% male)		68.4% (IQR 61.1–75.2)	50 (3624)	n/a	n/a	n/a
Modality (patients)	Venovenous	1177	54 (3808)	n/a		
	Venoarterial and combined	2508				
	Not specified	123				
ECMO duration (days)	All patients	8.2 (7.0–9.4)	49 (3328)	1781	97%	n/a
	Venoarterial patients only	5.6 (4.4–6.8)	20 (1895)	557	97%	<0.001
	Venovenous patients only	14.6 (10.6–18.6)	9 (309)	63	87%	

**Table 3:** Results—red cell transfusion rates.

Variable		Finding (95% Confidence Range)	Number of Papers (Patients) Included	Cochrane’s Q Test	I^2^ Test of Heterogeneity	*p*-Value for Comparison
ECMO Modality		PRBC Units/Run or PRBC Units/Day				
Whole ECMO run	All patients	17.7 (14.2–21.2)	52 (3452)	2816	98%	
	VA patients only	18.3 (14.2–22.4)	24 (2043)	1207	98%	0.85
	VV patients only	19.3 (10.4–28.1)	9 (309)	95	90%	
Per ECMO day	All patients	2.60 (1.93–3.27)	49 (3619)	3643	99%	
	VA patients only	3.86 (2.51–5.22)	23 (1933)	1519	99%	<0.001
	VV patients only	1.23 (0.89–1.57)	12 (665)	292	96%	
ECMO indication		PRBC units/day				
Postcardiotomy		5.56 (2.20–8.93)	8 (1078)	1235	99%	0.04
Nonsurgical		1.93 (1.26–2.59)	25 (1309)	730	97%	
Peripheral cannulation rate		PRBC units/day				
Greater than 90%		1.74 (1.24–2.25)	29 (2031)	1223	98%	0.02
Less than 90%		4.53 (2.31–6.76)	13 (1220)	793	99%	
Membrane type		PRBC units/day				
Polymethylpentene only		2.11 (1.49–2.73)	32 (2113)	1643	98%	0.11
Silicon, polypropylene or mixed		4.46 (1.68–7.24)	11 (895)	578	98%	
Survival status (median 51.2%)		PRBC units/day				
Above median		1.65 (1.08–2.23)	19 (1295)	965	98%	0.001
Below median		3.82 (2.23–5.42)	19 (1565)	1417	99%	
Major bleeding (median 30%)		PRBC units/day				
Above median		1.83 (1.14–2.52)	7 (336)	137	96%	0.99
Below median		1.84 (0.90–2.78)	8 (290)	210	97%	
Upper aPTT target (median 60s)		PRBC units/day				
Above median		2.76 (1.87–3.65)	8 (585)	115	94%	0.34
Below median		1.98 (0.64–3.32)	11 (1164)	409	98%	
Upper ACT target (median 180s)		PRBC units/day				
Above median		2.87 (1.57–4.16)	8 (343)	602	99%	0.92
Below and including median		2.95 (2.02–3.88)	14 (842)	301	96%	
Transfusion trigger (median 9.3 g/dL)		PRBC units/day				
Above and including median		2.39 (1.67–3.10)	15 (986)	758	98%	0.005
Below median		1.41 (0.86–1.97)	13 (797)	388	97%	

PRBC: units of packed red blood cells, VA—venoarterial, VV—venovenous.

## Supplementary Materials

The following are available online at https://www.mdpi.com/article/10.3390/membranes11040251/s1. Table S1: Search strategy. Table S2: Newcastle-Ottawa scores.
