# Supplementary material for: A Systematic Literature Review of Packed Red Cell Transfusion Usage in Adult Extracorporeal Membrane Oxygenation
Source: Membranes (Basel). 2021 Mar 30;11(4):251. doi: 10.3390/membranes11040251 (PMC8065680; doi:10.3390/membranes11040251)
Supplement: Supplementary file 1 [file membranes-11-00251-s001.pdf]

*membranes*

MDPI

*Review*

# A Systematic Literature Review of Packed Red Cell Transfusion Usage in Adult Extracorporeal Membrane Oxygenation

**Thomas Hughes [1], David Zhang [2], Priya Nair [1,2] and Hergen Buscher [1,2,*]**

[1] Department of Intensive Care Medicine, St Vincent's Hospital, Sydney 2010, Australia; thomas.hughes@health.nsw.gov.au (T.H.); Priya.Nair@svha.org.au (P.N.)
[2] Faculty of Medicine, University of New South Wales, Sydney 2052, Australia; davidzhang549@gmail.com
* Correspondence: Hergen.buscher@svha.org.au

**Table S1.** Search strategy.

| Medline |
|---|
| 1. exp extracorporeal oxygenation/ |
| 2. exp bleeding/ |
| 3. exp blood transfusion/ or exp transfusion medicine/ or exp transfusion/ or exp erythrocyte transfusion/ or exp plasma transfusion/ |
| 4. exp fresh frozen plasma/ |
| 5. exp fibrinogen/ |
| 6. exp heparin/ |
| 7. exp anticoagulation/ |
| 8. exp thromboembolism/ or exp venous thromboembolism/ |
| 9. exp lung embolism. |
| 10. 2 or 3 or 4 or 5 or 6 or 7 or 8 or 9. |
| 11. 1 and 10. |
| 12. limit 11 to (abstracts and human and (adult <18–64 years> or aged <65+ years)). |
| 13. limit 12 to yr =”1996–2016” |
| **Embase** |
| 1. exp extracorporeal membrane oxygenation/ |
| 2. exp hemorrhage/ |
| 3. exp platelet transfusion/ or blood transfusion/ or exp blood component transfusion/ or exp erythrocyte transfusion/ or exp transfusion medicine/ |
| 4. exp plasma/ |
| 5. exp fibrinogen/ |
| 6. exp heparin/ |
| 7. exp anticoagulants/ or exp thromboembolism/ |
| 8. exp pulmonary embolism/ |
| 9. 2 or 3 or 4 or 5 or 6 or 7 or 8. |
| 10. 1 and 9. |
| 11. limit 10 to (abstracts and English language and humans and “all adult (19 plus years)”) |

**Table S2.** Newcastle-Ottawa Scores.

| Author/Year | Type | Selection: Representativeness of Exposed Cohort | Selection: Respresentativeness of Non-Exposed Cohort | Selection: Ascertainment of Exposure | Selection: Demonstration that Out-Come Not Present at Study Start | Comparability of Cohorts Based on Design and Analysis | Outcome-Assessment | Out-Come-Timing of Follow Up | Out-Come-Adequacy of Follow Up | Total NOS (max = 9) |
|---|---|---|---|---|---|---|---|---|---|---|
| Anselmi 2016 | Cohort | | ✓ | | ✓ | ✓✓ | ✓ | ✓ | ✓ | 7 |
| Buscher 2016 | Cohort | ✓ | ✓ | ✓ | ✓ | ✓ | ✓ | ✓ | ✓ | 8 |
| Czobor 2016 | Cohort | ✓ | ✓ | ✓ | ✓ | ✓ | ✓ | ✓ | ✓ | 8 |
| Hryniewicz 2016 | Cohort | ✓ | | ✓ | ✓ | | ✓ | ✓ | ✓ | 6 |
| Krueger 2016 | Cohort | ✓ | | ✓ | ✓ | | ✓ | ✓ | ✓ | 6 |
| Mazzeffi 2016 | Cohort | ✓ | ✓ | ✓ | ✓ | ✓ | ✓ | ✓ | ✓ | 8 |
| Opfermann 2016 | Cohort | ✓ | ✓ | ✓ | ✓ | ✓ | ✓ | ✓ | ✓ | 8 |
| Pan 2016 | Cohort | ✓ | ✓ | ✓ | ✓ | | ✓ | ✓ | ✓ | 7 |
| Staudacher 2016 | Cohort | ✓ | ✓ | ✓ | ✓ | ✓ | ✓ | ✓ | ✓ | 8 |
| Tanaka 2016 | Cohort | ✓ | ✓ | ✓ | ✓ | ✓ | ✓ | ✓ | ✓ | 8 |
| Tauber 2016 | Cohort | ✓ | ✓ | ✓ | ✓ | ✓ | ✓ | ✓ | ✓ | 8 |
| Trudzinski 2016 | Cohort | ✓ | ✓ | ✓ | | | ✓ | ✓ | | 5 |
| Agerstrand 2015 | Cohort | ✓ | ✓ | ✓ | ✓ | ✓ | ✓ | ✓ | ✓ | 8 |
| Esper 2015 | Cohort | ✓ | | ✓ | ✓ | | ✓ | ✓ | ✓ | 6 |
| Halaweish 2015 | Cohort | ✓ | ✓ | ✓ | ✓ | | ✓ | ✓ | ✓ | 7 |
| Ius 2015 | Cohort | ✓ | | ✓ | ✓ | | ✓ | ✓ | ✓ | 6 |
| Lehle 2015 | Cohort | ✓ | ✓ | ✓ | ✓ | ✓✓ | ✓ | ✓ | ✓ | 9 |
| Li 2015 | Cohort | ✓ | ✓ | ✓ | | ✓ | ✓ | ✓ | ✓ | 7 |
| Mohite 2015 | Cohort | ✓ | ✓ | ✓ | ✓ | | ✓ | ✓ | ✓ | 7 |
| Omar 2015 | Cohort | ✓ | ✓ | ✓ | ✓ | ✓ | ✓ | ✓ | ✓ | 8 |
| Panigada 2015 | Cohort | ✓ | | ✓ | ✓ | | ✓ | ✓ | ✓ | 6 |
| Poss 2015 | Cohort | | | ✓ | ✓ | | ✓ | ✓ | ✓ | 5 |
| San Roman 2015 | Cohort | ✓ | | ✓ | ✓ | | ✓ | ✓ | ✓ | 6 |
| Voelker 2015 | Cohort | ✓ | ✓ | ✓ | ✓ | | ✓ | ✓ | ✓ | 7 |
| Wu 2015 | Cohort | ✓ | | ✓ | ✓ | | ✓ | ✓ | ✓ | 6 |
| Guirand 2014 | Cohort | ✓ | ✓ | ✓ | ✓ | | ✓ | ✓ | ✓ | 7 |
| Loforte 2014 | Cohort | ✓ | ✓ | ✓ | ✓ | | ✓ | ✓ | ✓ | 7 |
| Roch 2014 | Cohort | ✓ | ✓ | ✓ | ✓ | | ✓ | ✓ | ✓ | 7 |
| Shum 2014 | Cohort | ✓ | ✓ | ✓ | ✓ | | ✓ | ✓ | ✓ | 7 |
| Fagnoul 2013 | Cohort | ✓ | ✓ | ✓ | ✓ | | ✓ | ✓ | ✓ | 7 |
| Michaels 2013 | Cohort | ✓ | ✓ | ✓ | ✓ | | ✓ | ✓ | ✓ | 7 |
| Mikus 2013 | Cohort | ✓ | ✓ | ✓ | ✓ | | ✓ | ✓ | ✓ | 7 |
| Pieri 2013 | Cohort | ✓ | ✓ | ✓ | ✓ | | ✓ | ✓ | ✓ | 7 |
| Repesse 2013 | Cohort | | ✓ | ✓ | ✓ | | ✓ | ✓ | ✓ | 6 |
| Loforte 2012 | Cohort | | ✓ | ✓ | ✓ | | ✓ | ✓ | ✓ | 6 |
| Park 2012 | Cohort | ✓ | ✓ | ✓ | ✓ | | ✓ | ✓ | ✓ | 7 |
| Garcia 2011 | Cohort | | ✓ | ✓ | ✓ | | ✓ | ✓ | ✓ | 6 |
| Han 2011 | Cohort | ✓ | ✓ | ✓ | ✓ | | ✓ | ✓ | ✓ | 7 |
| Lamarche 2011 | Cohort | | | ✓ | ✓ | | ✓ | ✓ | ✓ | 5 |
| Formica 2010 | Cohort | ✓ | ✓ | ✓ | ✓ | | ✓ | ✓ | ✓ | 7 |
| Kanji 2010 | Cohort | ✓ | ✓ | ✓ | ✓ | | ✓ | ✓ | ✓ | 7 |
| Marasco 2010 | Cohort | ✓ | ✓ | ✓ | ✓ | | ✓ | ✓ | ✓ | 7 |
| Rastan 2010 | Cohort | ✓ | ✓ | ✓ | ✓ | | ✓ | ✓ | ✓ | 7 |
| Ang 2009 | Cohort | ✓ | ✓ | ✓ | ✓ | | ✓ | ✓ | ✓ | 7 |
| Davies 2009 | Cohort | ✓ | ✓ | ✓ | ✓ | | ✓ | ✓ | | 6 |
| Muller 2009 | Cohort | ✓ | ✓ | ✓ | ✓ | | ✓ | ✓ | ✓ | 7 |
| Bakhtiary 2008 | Cohort | ✓ | ✓ | ✓ | ✓ | | ✓ | ✓ | ✓ | 7 |
| Dietl 2008 | Cohort | ✓ | ✓ | ✓ | ✓ | | ✓ | ✓ | ✓ | 7 |

| | | | | | | | | | | |
|---|---|---|---|---|---|---|---|---|---|---|
| Frencker 2002 | Cohort | ✓ | ✓ | ✓ | ✓ | | ✓ | ✓ | ✓ | 7 |
| Smith 2001 | Cohort | ✓ | ✓ | ✓ | ✓ | | ✓ | ✓ | ✓ | 7 |
| Lewandowski 1997 | Cohort | | | ✓ | ✓ | | ✓ | ✓ | ✓ | 5 |
| Peek 1997 | Cohort | ✓ | ✓ | ✓ | ✓ | | ✓ | ✓ | ✓ | 7 |
| Butch 1996 | Cohort | ✓ | ✓ | ✓ | ✓ | | ✓ | ✓ | ✓ | 7 |
| Muehrcke 1996 | Cohort | ✓ | ✓ | ✓ | ✓ | | ✓ | ✓ | ✓ | 7 |
